# Immune Checkpoint Inhibitor-Related Cytokine Release Syndrome: Analysis of WHO Global Pharmacovigilance Database

**DOI:** 10.3389/fphar.2020.00557

**Published:** 2020-05-04

**Authors:** Alessandro Ceschi, Roberta Noseda, Karine Palin, Katia Verhamme

**Affiliations:** ^1^Division of Clinical Pharmacology and Toxicology, Institute of Pharmacological Sciences of Southern Switzerland, Ente Ospedaliero Cantonale, Lugano, Switzerland; ^2^Department of Clinical Pharmacology and Toxicology, University Hospital Zurich, Zurich, Switzerland; ^3^Faculty of Biomedical Sciences, University of Southern Switzerland, Lugano, Switzerland; ^4^Université de Bordeaux, Collège Santé, Bordeaux, France; ^5^Department of Medical Informatics, EMC Rotterdam, Rotterdam, Netherlands

**Keywords:** immune checkpoint inhibitors, cytokine release syndrome, VigiBase, adverse drug reaction, pharmacovigilance

## Abstract

Immune checkpoint inhibitors (ICIs) have proven effective in the treatment of numerous cancers; however, they have been associated with immune-related adverse events (irAEs), among which cytokine release syndrome (CRS) has been reported in a few case reports. To describe the burden of ICI-related CRS and raise awareness of CRS as irAE, we queried VigiBase, the World Health Organization global database of spontaneously reported suspected adverse drug reactions (ADRs), and retrieved safety reports of suspected CRS associated with ICIs, gathered in the database through January 12^th^ 2020. We assessed ICI-related CRS safety reports in terms of geographical and temporal patterns of reporting, patient demographics and clinical features, treatment characteristics, CRS clinical presentation, timing, seriousness, and outcome. We retrieved 58 cases of whom 43 (74%) reported CRS with anti-programmed death-1/anti-programmed death-ligand 1 agents. Melanoma (n=17, 29%) and hematologic malignancies (n=16, 28%) were the most common underlying cancers. ICIs were the solely suspected drugs in 37 (64%) cases. Typical signs and symptoms of CRS were reported in 25 (43%) patients. ICI-related CRS developed a median of 4 weeks after ICI initiation (IQR 1–18 weeks, n=9, 16%). Besides two fatal cases, CRS recovered/was recovering at the time of reporting in 35 (60%) cases. We observed differences in the geographical pattern of ICI-related CRS reporting, with a high proportion of ICI-related CRS cases in Australia and North America (0.14 and 0.10% respectively). Due to ICI expanding indications, clinicians should be aware that ICIs could contribute to CRS onset in cancer patients as pharmacological triggers.

## Introduction

Immune checkpoint inhibitors (ICIs) have proven effective in the treatment of numerous cancers (Ribas and Wolchok, 2018). By inhibiting negative regulators of the immune system, ICIs enhance immune activity against cancer cells and, aberrantly, against host non-cancer cells, resulting in inflammatory side effects also called immune-related adverse events (irAEs) (Postow et al., 2018).

Cytokine release syndrome (CRS) is a systemic inflammatory disease characterized by a massive release of cytokines (Gödel et al., 2018). It can present with a variety of symptoms ranging from mild to life threatening, being sometimes fatal (Lee et al., 2014). Mild symptoms of CRS include fever, fatigue, nausea, vomiting, headache, rash, arthralgia, myalgia, and malaise. In most severe cases, hypotension requiring high-dose vasopressors and hypoxia requiring mechanical ventilation may occur as life-threating complications. Moreover, laboratory abnormalities that are common in patients with CRS include cytopenias, coagulopathy, elevated liver enzymes and creatinine, and high C reactive protein levels (Shimabukuro-Vornhagen et al., 2018). CRS can be triggered by infections or be associated with drugs such as monoclonal antibodies (e.g., rituximab), conventional chemotherapy, and immunotherapies with chimeric antigen receptor T (CAR T) cells (Shimabukuro-Vornhagen et al., 2018).

Despite the strict parallelism between ICI mechanism of action and CRS pathophysiologic mechanism, to date, only a few case reports have documented the association of ICIs as pharmacological triggers of CRS in cancer patients (Foran et al., 2017; Rassy et al., 2017; Rotz et al., 2017; Zhao et al., 2018; Dimitriou et al., 2019; Honjo et al., 2019; Kogure et al., 2019; Oda et al., 2019).

To describe the burden of ICI-related CRS and raise awareness of CRS as irAE, we retrieved from VigiBase, the World Health Organization (WHO) global database of spontaneously reported suspected adverse drug reactions (ADRs), the largest-to-date series of ICI-related CRS safety reports and assessed the geographical and temporal patterns of reporting, patient demographics and clinical features, treatment characteristics, CRS clinical presentation, timing, seriousness, and outcome.

## Methods

VigiBase (http://www.vigiaccess.org/), developed and maintained by the WHO Collaborating Centre for International Drug Monitoring, Uppsala Monitoring Centre (WHO-UMC), gathers over 20 million anonymized safety reports of suspected ADRs spontaneously reported by physicians, pharmacists, health professional, pharmaceutical companies and patients, from over 130 member countries of the WHO Program for International Drug Monitoring. VigiBase facilitates the detection of drug safety signals, highlights rare ADRs and, through a continuous monitoring, can capture delayed ADRs. Safety reports include administrative information (country of origin, reporting date, seriousness), patient demographics (age at ADR onset and sex), indication, start and end dates of reported drugs, ADRs with onset dates and outcomes. The Medical Dictionary for Regulatory Activities (MedDRA) allows for ADRs’ classification according to hierarchical groups, whereby “preferred terms” (PTs) describe ADRs as single medical concepts.

We performed a retrospective observational study and retrieved from VigiBase safety reports associated with ipilimumab (of the anatomical therapeutic chemical, ATC, class L01XC11), nivolumab (L01XC17), pembrolizumab (L01XC18), atezolizumab (L01XC32), avelumab (L01XC31), durvalumab (L01XC28), and cemiplimab (L01XC33), whereby the role of the administered ICI was indicated by reporter as “suspected” for the adverse event(s). Subsequently, among ICI-related safety reports, we selected those reporting as adverse event CRS, defined accordingly to the correspondent MedDRA PT “cytokine release syndrome” (MedDRA version 21.1). The fact that reporters defined the ICI role as “suspected” in CRS onset (and ruled out either an “interacting” or a “concomitant” role), was *per se* the assumption for classifying the safety reports as exposed, even if these did not explicitly record the start and end dates for the ICI treatment as a confirmation. Safety report retrieval ranged from database inception through January 12^th^ 2020.

Based on molecular targets, we distinguished anti-cytotoxic T-lymphocyte antigen-4 agents (anti-CTLA-4, i.e., ipilimumab), from anti-programmed death-1/programmed death-Ligand 1 (anti-PD-1/PD-L1, i.e., pembrolizumab, nivolumab, atezolizumab, avelumab durvalumab, and cemiplimab).

We assessed ICI-related CRS safety reports in terms of geographical and temporal patterns of reporting; patient age and sex; cancer type; treatment characteristics (ICI drug and regimen; co-suspected drugs; duration); CRS clinical presentation, timing, seriousness, and outcome. We summarized categorical variables using frequency and percentage, continuous variables using median, and interquartile range (IQR). Analyses were carried out by Microsoft Excel (2010, Microsoft Corporation, Washington, USA).

According to the Human Research Act (810.30, of September 30^th^, 2011—status as of January 1st, 2014), from the Federal Assembly of the Swiss Confederation, ethical approval was not required (Art. 2: “It does not apply to research which involves anonymously collected or anonymised health-related data”).

## Results

As of January 12^th^ 2020, VigiBase gathered 80,700 safety reports of ICI-related ADRs, among which 58 concerned CRS. The geographical pattern of ICI-related CRS reporting varied across continents. In Australia the proportion of ICI-related CRS cases out of the total number of ICI-related safety reports was the highest (3/2,126, 0.14%), followed by North America (United States and Canada; 37/36,500, 0.10%). Conversely, ICI-related CRS reporting was lower in Europe (12/18,756, 0.06%) and in Japan (6/13,267, 0.05%). **Table 1** summarizes baseline characteristics of ICI-related CRS safety reports. ICI-related CRS reporting increased over time, with 27 cases (47%) in 2019 (no cases of ICI-related CRS reported in 2020 as of January 12^th^). Melanoma (n=17, 29%) and hematologic malignancies (n=16, 28%) were the most common underlying cancers. Regardless of cancer type, ICI-related CRS safety reports were more numerous for anti-PD-1/PD-L1 antibodies (43, 74%). ICIs were the solely suspected drugs in 37 (64%) safety reports. Among the other 21 (36%) safety reports with additional suspected drugs, 18 (31%) listed antineoplastic agents. Concurrent infections were reported in six (10%) patients, with pneumonia (n=2), urinary tract infection/cystitis (n=1), upper respiratory tract infection/sinusitis (n=1), *Staphylococcal bacteraemia*/*Pseudomonas* infection (n=1), and sepsis (n=1). CRS was the solely reported ICI-related ADR in 21 (36%) patients. Among the other 37 (64%) safety reports with additional ADRs co-reported with CRS, 25 (43%) listed ADRs that are typical signs and symptoms of CRS (**Table 2**). ICI-related CRS developed a median of four weeks after ICI initiation (IQR 1–18 weeks, n=9, 16%). Except for one case of unknown seriousness and two cases (3%) recorded as not serious, reporters evaluated ICI-related CRS cases as serious in 55 (95%) patients. In 25 (43%) cases, ICI-related CRS caused or prolonged hospitalization, in 10 (17%) it was life threatening, and in 18 (31%), CRS determined a clinically relevant condition not further specified. There were two fatal cases, whereby CRS contributed to worsening the condition of the patients along with infections and tumor progression. Beside these latter cases, CRS either recovered or was recovering at the time of reporting in 35 (60%) cases. In 20 (34%) safety reports, CRS outcome was unknown and one patient recovered from ICI-related CRS with sequelae.

**Table 1:** Characteristics of safety reports of immune checkpoint inhibitor-related cytokine release syndrome.

Characteristics of safety reports	n (%) (N=58)
Reporting year	
2015	5 (9)
2016	3 (5)
2017	6 (10)
2018	17 (29)
2019	27 (47)
Age	
Reported	50 (86)
Median [IQR], years	55 [44–68]
Not reported	8 (14)
Sex	
Male	34 (59)
Female	21 (36)
Not reported	3 (5)
Cancer type	
Melanoma	17 (29)
Hematologic malignancy^a^	16 (28)
Lung cancer^b^	11 (19)
Other^c^	6 (10)
Not reported	8 (14)
Regimen	
Anti-CTLA-4 (ipilimumab) monotherapy	3 (5)
Anti-PD-1 monotherapy	
Nivolumab	21 (36)
Pembrolizumab	12 (21)
Cemiplimab	1 (2)
Anti-PD-L1 monotherapy	
Atezolizumab	7 (12)
Avelumab	2 (3)
Nivolumab and ipilimumab combination therapy	7 (12)
Nivolumab and ipilimumab treatment not definable^d^	2 (3)
Pembrolizumab and ipilimumab treatment not definable^d^	2 (3)
Pembrolizumab, followed by nivolumab and ipilimumab combination	1 (2)
Duration	
Single administration	10 (17)
Prolonged	10 (17)
Median [IQR], weeks	11 [4–24]
More than 1 year	1 (2)
Not definable^d^	37 (64)
Co-suspected drugs	
Not reported	37 (64)
Reported	21 (36)
Antineoplastic agents^e^	18
Other^f^	4

IQR, interquartile range; CTLA-4, cytotoxic T-lymphocyte antigen 4; PD-1, programmed cell death protein 1; PD-L1, programmed cell death-ligand 1.

a Hodgkin disease (n=5); diffuse large B-cell lymphoma refractory (n=4); diffuse large B-cell lymphoma (n=2); lymphoma, non-Hodgkin’s lymphoma refractory, acute myeloid leukemia, primary mediastinal large B-cell lymphoma, acute lymphocytic leukemia (all n=1).

b Non-small cell lung cancer (n=5); adenocarcinoma of the lung (n=3); pulmonary carcinoma, squamous cell carcinoma of the lung, lung neoplasm malignant (all n=1).

c Alveolar soft part sarcoma (n=2); breast cancer metastatic, gastric cancer, squamous cell carcinoma of skin, triple negative breast cancer (all n=1).

d Because of partially recorded or missing ICI start and/or end dates.

e Axicabtagene ciloleucel, cyclophosphamide, fludarabine (n=4); brentuximab vedotin, cobimetinib, vemurafenib (n=2); azacitidine, blinatumomab, carboplatin, dabrafenib, decitabine, IMCgp100, NY-ESO-1, pazopanib, pemetrexed, siltuximab, talimogene laherparepvec, tisagenlecleucel, trametinib (all n=1). Some safety reports had multiple co-suspected antineoplastic agents.

f Lisinopril and morphine (n=1); mesna, naloxegol, prednisolone (all n=1).

**Table 2:** Co-reported clinical signs and symptoms of immune checkpoint inhibitor-related cytokine release syndrome.

Co-reported clinical signs and symptoms of CRS	Number of safety reports n (%)^a^, N=58
Constitutional	13 (22)
Fatigue	2
Pyrexia	8
Asthenia	1
Malaise	1
Myalgia	2
Arthralgia	2
Skin	4 (7)
Rash	2
Rash maculo-papular	2
Gastrointestinal	6 (10)
Diarrhea	4
Nausea	3
Respiratory	6 (10)
Acute respiratory distress syndrome	2
Respiratory failure	2
Pulmonary edema	1
Pleural effusion	3
Hypoxia	1
Cardiovascular	4 (7)
Tachycardia	2
Hypotension	3
Renal	6 (10)
Acute kidney injury	3
Nephritis	1
Renal failure	2
Neurologic	4 (7)
Headache	2
Hypotonia	1
Tremor	1
Laboratory abnormalities	3 (5)
Anemia	1
Platelet count decreased	2
Neutrophil count decreased	1
Lactic acidosis	1
Thrombocytopenia	1
Leukopenia	1
Neutropenia	1
Lymphopenia	1
Coagulation	1 (2)
Disseminated intravascular coagulation	1

CRS, cytokine release syndrome.

a Some patients reported multiple signs and symptoms of cytokine release syndrome.

## Discussion

By analyzing the WHO global pharmacovigilance database, we described the largest-to-date series of CRS cases reported in association with ICI treatment.

By hyper-activating the immune system to overcome the inhibitory signals of cancer cells, ICIs can provoke on-target autoimmune toxicity (when the targeted tumor antigen is on host non-cancer cells), as well as off-target cytokine-associated toxicity (also known as CRS) (Lee et al., 2014). In CRS, T cells, B cells, natural killer cells, macrophages and endothelial cells, release a variety of cytokines, among which interleukin-6 (IL-6) plays a central role (Lee et al., 2014). Noteworthy, functional genetic variants in *IL-6* gene have been identified, which lead to IL-6 overexpression (Fishman et al., 1998). On this basis, the geographical pattern that we observed in the reporting of ICI-related CRS, which was highest in Australia and lowest in Japan, might suggest that polymorphisms in *IL-6* gene could predispose patients to develop CRS upon treatment with ICIs. However, such a variability could also depend on the existence of differences among reporting countries concerning the awareness, recognition, and the extent of spontaneous reporting of CRS as ICI-related ADR.

ICI-related CRS reporting peaked in 2019 suggesting that, albeit likely depending on the progressively increasing use of ICIs for a variety of cancer types, CRS is progressively more easily recognized and diagnosed as associated to ICIs. Accordingly, among the limited isolated case reports published so far on CRS onset during treatment with ICIs, the majority deals with very recent cases, on either pembrolizumab or nivolumab used to treat different cancer types (Dimitriou et al., 2019; Honjo et al., 2019; Kogure et al., 2019; Oda et al., 2019).

CRS represents one of the most frequent serious adverse effects of bispecific antibody constructs and CAR T cell therapies recently approved for hematologic malignancies, such as acute lymphoblastic B cell leukemia, chronic lymphocytic leukemia, and diffuse large B cell lymphoma (June and Sadelain, 2018). Consistently, we observed that the tumor-specific pattern of CRS onset on ICIs involved more commonly patients with hematological malignancies. Moreover, a considerable number of ICI-related CRS cases was reported in patients with melanoma, probably because of the earlier approval of ICIs for this indication (Postow et al., 2015; Hodi et al., 2018).

Regardless of cancer type, ICI-related CRS safety reports were more numerous on anti-PD-1/PD-L1 antibodies, in line with the single case reports of CRS developed on either nivolumab or pembrolizumab (Foran et al., 2017; Rassy et al., 2017; Rotz et al., 2017; Zhao et al., 2018; Dimitriou et al., 2019; Honjo et al., 2019; Kogure et al., 2019; Oda et al., 2019). Moreover, a recent review of the largest clinical trials of ICIs (Michot et al., 2019), found that the frequency of hematological irAEs of all grades (including CRS), was higher with anti-PD-1 (4.1%) and anti-PD-L1 (4.7%) than with anti-CTLA-4 agents (0.5%, *P* < 0.0001). However, our finding might also reflect a more widespread use of anti-PD-1/PD-L1 agents in clinical practice as compared to anti-CTLA-4 (Khoja et al., 2017).

Noteworthy, beyond ICIs, co-suspected drugs were absent in a remarkable proportion of safety reports, suggesting that ICIs could contribute to CRS onset as pharmacological triggers. When otherwise present, co-suspected drugs were mostly antineoplastic agents known from either clinical studies or previous case reports for causing CRS. Among these, the bispecific antibody IMCgp100, the CAR T cell therapy tisagenlecleucel and the antibody-drug conjugate that targets CD30, brentuximab vedotin (Alig et al., 2015; Frey and Porter, 2016).

Seven patients reported as co-suspected drugs chemotherapeutic agents. Although the correspondent safety reports lacked information on the timing of chemotherapy administration (which could have been antecedent or concomitant with ICI treatment), the associated lymphocyte-depletion could have affected the risk of CRS development. Indeed, a higher incidence of CRS was observed after lymphocyte-depletion with cyclophosphamide or fludarabine in patients treated with CD19 CAR T cells (Hay et al., 2017). Although CRS has been described in the setting of bacterial or viral infections (Shimabukuro-Vornhagen et al., 2018), among the cases that we analyzed, infections were reported in only six patients.

Since CRS is clinically defined by a constellation of inflammatory symptoms and signs, the majority of which is non-specific, its diagnosis can be difficult (Gödel et al., 2018). Temporal correlation and some abnormal laboratory findings (e.g., increased creatinine, increased liver enzymes, and altered coagulation parameters) represent crucial diagnostic hints of CRS (Gödel et al., 2018). However, we could not properly evaluate patients’ laboratory tests, due to the scant clinical richness of safety reports. Conversely, we described that ICI-related CRS developed with a median of 4 weeks since ICI initiation, which widely fits within the 10-week mean time to onset described for ICI-related hematological ADRs by Michot et al. (2019) in the resume of 63 case reports among which 7 of ICI-related CRS.

We observed that ICI-related CRS either recovered or was recovering at the time of reporting in most cases, with only two fatal outcomes.

With an exploratory intent and the purpose of generating hypotheses to be potentially tested and confirmed in future studies, on larger datasets and through different data sources and study designs, we assessed the impact of some patient and ICI treatment characteristics on CRS outcome (**Supplementary Table 1**). We observed that CRS resolution occurred more frequently in association with female sex. Female sex as a factor that possibly prevents cancer patients from developing severe ICI-related CRS could rely on the existence of differences in the immune response between males and females (Klein and Flanagan, 2016). However, if efficacy of ICI treatment in advanced cancers was shown not to be affected by patient sex, data on the impact of sex on ICI-related adverse event rate in general are still lacking (Özdemir et al., 2018). Moreover, the reporting frequency of ICI-related recovered/recovering CRS increased in association with younger age, ICI monotherapy rather than the combination, and prolonged treatments (although without reaching statistical significance probably due to small sample size). We highlighted that younger age (defined as <65 years in the present study) might be more frequently associated with ICI-related CRS positive outcome. This observation, whenever confirmed on a larger number of patients, would suggest that older adults (aged ≥65 years) could be exposed to a higher risk of developing severe CRS, in contrast to the general knowledge of comparable safety profiles of ICIs regardless of patient age (Betof et al., 2017). ICI-related CRS having more frequently a positive outcome in association with ICI monotherapy, confirmed the higher toxicity—more severe and with an earlier onset—of ICI combination as compared to single agents (Hodi et al., 2018). Lastly, the number of ICI-related CRS events with a positive outcome was higher with ICI prolonged treatments, thus supporting former findings about the most serious ICI-related toxicities generally occurring following ICI single administration (Sznol et al., 2017)

Relying on a spontaneous reporting system, VigiBase suffers from some general limitations that include reporting bias (e.g., underreporting and selective reporting) and missing data. With regard to co-reported drugs, it is important to mention that in VigiBase, despite there being no other suspected, interacting, or concomitant drugs specified in the report, other drugs might have been taken by the patient, without being considered sufficiently relevant by reporter to be recorded or were omitted for unknown reasons. Therefore, assessing a causal relationship between a drug and an adverse event is complex and benefits from having the more information available. As safety reports derive from a variety of sources, they contain heterogeneous information and are rarely sufficient to confirm that a particular drug caused the ADR in a specific patient. Moreover, safety reports do not provide information on the number and demographics as well as clinical characteristics of patients exposed to the drug but who did not develop ADRs (Lindquist, 2008). Further specific limitations concern the small sample size and the fact that, being CRS a systemic inflammatory condition, a variety of other factors such as infections and other drugs could have triggered it in addition to ICIs (Shimabukuro-Vornhagen et al., 2018). Lastly, because of exclusion from the present case series of safety reports with signs and/or symptoms of CRS reported as separate adverse events on ICIs, without CRS as preferred term, we could have underestimated the number of safety reports associated with ICI(s) concerning suspected CRS.

## Conclusions

Due to ICI expanding indications, clinicians should be aware that ICIs could contribute to CRS onset in cancer patients as pharmacological triggers. Further studies are needed to better characterize the mechanisms underlying ICI-related CRS.

## Data Availability Statement

Datasets are available on request: The raw data supporting the conclusions of this manuscript will be made available by the authors, without undue reservation, to any qualified researcher.

## Ethics Statement

Ethical review and approval was not required for the study on human participants in accordance with the local legislation and institutional requirements. Written informed consent for participation was not required for this study in accordance with the national legislation and the institutional requirements.

## Author Contributions

AC and KV designed the study. AC and RN did the literature search, and collected and analyzed the data. All authors contributed to the interpretation of the data. AC and RN wrote the manuscript and all authors read and approved the final version of the manuscript.

## Conflict of Interest

The authors declare that the research was conducted in the absence of any commercial or financial relationships that could be construed as a potential conflict of interest.
