# Supplementary material for: Immune Checkpoint Inhibitor-Related Cytokine Release Syndrome: Analysis of WHO Global Pharmacovigilance Database
Source: Front Pharmacol. 2020 May 4;11:557. doi: 10.3389/fphar.2020.00557 (PMC7212758; doi:10.3389/fphar.2020.00557)
Supplement: Supplementary file 1 [file Table_1.docx]

**Supplementary Table 1** Impact of patient sex, age, and cancer type, and of immune checkpoint inhibitor drug, regimen, and treatment duration, on the reporting frequency of recovered/recovering cytokine release syndrome.

|  | CRS recovered/recovering  n/N (%) | *P* value* |
| --- | --- | --- |
| Patient sex  Male  Female | 17/34 (50)  18/21 (86) | 0.01 |
| Patient age  <65 years  ≥65 years | 23/33 (70)  7/17 (41) | 0.099 |
| Cancer type  Melanoma  Lung cancer | 7/13 (54)  6/11 (55) | >0.1 |
| ICI drug  Anti-CTLA-4  Anti-PD-1/PD-L1 | 2/3 (67)  29/43 (67) | >0.999 |
| ICI regimen  Monotherapy  Combination | 31/46 (67)  3/7 (43) | >0.1 |
| Treatment duration  Single administration  Prolonged | 7/10 (70)  9/11 (82) | >0.1 |

Abbreviations: CRS Cytokine Release Syndrome; ICI Immune Checkpoint Inhibitor; CTLA-4 Cytotoxic T-Lymphocyte Antigen 4; PD-1 Programmed cell Death protein 1; PD-L1 Programmed cell Death-Ligand 1

* Chi-square statistical test (with the Yates’ correction when appropriate or the Fisher’s exact test for expected cell counts less than five). *P* values considered significant when <0.05. Analyses performed with GraphPad Prism 8 (GraphPad Software Inc., San Diego, USA).
